# Factors Associated With Digital Health Literacy in the United Kingdom: Cross-Sectional Online Survey

**DOI:** 10.2196/89136

**Published:** 2026-07-08

**Authors:** Holly Exton-Smith, Sara Sousi, Albandari Alharbi, Austen El-Osta, Geva Greenfield, Benedict Hayhoe, Azeem Majeed, Ana Luísa Neves

**Affiliations:** 1 Department of Primary Care and Public Health, School of Public Health, Imperial College London, 90 Wood Lane, London, England, W12 0BZ, United Kingdom, 44 020 7589 5111; 2 Self-Care Academic Research Unit (SCARU), Department of Primary Care and Public Health, Imperial College London, London, England, United Kingdom

**Keywords:** digital health, eHealth literacy, health inequalities, cross-sectional studies, online survey, digital health literacy, eHealth Literacy Scale, equity, digital tools

## Abstract

**Background:**

Digital health literacy (DHL), the ability to seek, understand, and apply digital health information, is increasingly important in the United Kingdom, with a focus on digital transformation within the health service. While digital tools offer potential to improve access and equity, they may exacerbate existing health inequities if segments of the population are unable to engage with them effectively. Understanding the sociodemographic, economic, and social factors associated with DHL is essential to designing inclusive digital health services.

**Objective:**

This study aimed to measure DHL among UK adults and identify its sociodemographic, economic, and social associates.

**Methods:**

A cross-sectional online survey was disseminated to a nationally representative sample of UK adult internet users from November to December 2024. DHL was self-reported using the validated eHealth Literacy Scale (eHEALS), which ranges from 8 to 40. eHEALS score was dichotomized into high and low DHL based on a cutoff of 26. Multivariable logistic regression was used to identify associates of DHL, with missing data handled using multiple imputation by chained equations.

**Results:**

The median eHEALS score was 31 (IQR 27‐32); 21% (320/1525) of the participants had low DHL, while 79% (1205/1525) had high DHL. Those aged 65 years and older, compared with those in the age group of 18‐44 years, had higher odds of low DHL (odds ratio [OR] 1.43, 95% CI 1.02‐2.01; *P*=.04). Those belonging to a lower social grade also had higher odds of low DHL, compared with those belonging to the higher social grade (OR 1.37, 95% CI 1.05‐1.80; *P*=.02). Females had lower odds of low DHL (OR 0.60, 95% CI 0.46‐0.77; *P*<.001), as did those with an undergraduate or postgraduate degree or higher, compared with those educated to below degree level (undergraduate degree OR 0.52, 95% CI 0.37‐0.74, *P*<.001; postgraduate degree or higher OR 0.58, 95% CI 0.40‐0.82, *P*=.002). Those who socialized daily, compared to those who did this never or rarely, had marginally lower odds of low DHL (OR 0.64, 95% CI 0.42‐1.00; *P*=.05). In subgroup analysis among participants with chronic health conditions, age and social grade were not significant associates of DHL.

**Conclusions:**

Among UK internet users, male sex, lower educational attainment, lower social grade, less frequent socializing, and older age were statistically significant associates of low DHL. The model’s modest explanatory power suggests that additional factors beyond those examined play an important role. As findings are based on internet users, the prevalence of low DHL in the general population is likely higher than reported. This study provides a partial basis for identifying groups who may benefit from additional support, but intervention design should not rely solely on factors identified here. Inclusive interventions accounting for a broader range of factors are needed to ensure that digital transformation in health care narrows rather than widens health inequities.

## Introduction

Digital health encompasses the use of digital technologies to improve health and health care delivery [1]. Early examples include telemedicine, online health information, and electronic health records, while wearable technologies for health (wearables), mobile device apps, online communities, and social media are more recent developments [2]. These tools are changing the way populations access health care and communicate with health care professionals, as well as how we monitor symptoms, manage diseases, and track our health more generally. The National Health Service (NHS) in the United Kingdom has a digital strategy, which includes digital general practice services and electronic prescriptions [3,4]. Based on findings from an investigation of the current state of the NHS in England [5], digital transformation is 1 of 3 shifts outlined in the recent 10 Year Health Plan for England [4].

Health literacy is widely recognized as a determinant of health outcomes and a major public health challenge [6-9]. Improved digital health literacy (DHL) is crucial for navigating digital health technologies and maximizing the benefits of modern health care [10,11]. DHL is defined as the ability to search, find, understand, and evaluate health information from electronic resources and to use the knowledge gained to solve health-related problems [12]. DHL can facilitate self-management of health [13,14], mitigate the effects of digital misinformation [15-17], and improve access to health care [18,19]. Several tools have been developed to measure DHL, with the most used being the eHealth Literacy Scale (eHEALS) [10,20].

eHEALS has been validated in a range of languages (including German and Dutch [21,22]), settings (including high- and low-income countries [22,23]), and social groups (including university students and older adults [14,24-26]). eHEALS has demonstrated good internal consistency, with Cronbach α values typically ranging from 0.88 to 0.93 across studies [21,27,28]. Norman and Skinner [28] found the tool to have a single-factor structure in their sample of adolescents; however, subsequent studies have found it to have a 1-, 2-, or 3-factor structure among different populations [22,29]. The tool’s brevity makes it popular in both research and clinical settings [30].

Given the importance of DHL, several studies have investigated its associates. However, most studies have focused on a specific population, and their ability to generalize findings to the UK population is limited [10,31]. In addition, studies have focused on a narrow scope of possible associates, potentially neglecting other factors that influence DHL. Despite these limitations, certain relationships have been identified in the literature. Older age is consistently found to be an associate of low DHL [10,32,33], while higher educational attainment and higher socioeconomic status have been found to positively affect DHL [10,31,33]. Other factors—such as urbanicity, ethnicity, primary language, employment status, and social support—are also possible determinants of DHL [10,33-36]. However, further investigation is warranted. Additionally, health conditions and self-perceived health status may influence DHL. A cross-European study found that self-reported health was a positive determinant of eHEALS score [32], and better self-reported health status has been shown to be associated with DHL skills, after adjusting for various socioeconomic factors and use of the internet for seeking health-related information [17]. Notably, respondents with a long-term disease also exhibited increased agreement with DHL-related skills [17]. In contrast, a meta-analysis across 7 studies found that participants with existing disease or at a higher risk for disease had lower eHEALS scores [31]. These findings highlight the challenge in parsing the influence of chronic health conditions compared with perceived health status [17,21,22].

The aim of this study was to measure DHL among adults in the United Kingdom and identify associated sociodemographic, economic, and social factors. A better understanding of both the DHL level and the factors associated with it in the United Kingdom will be critical to inform the design of more tailored digital health tools and targeted interventions. This ensures that those who need health information can access, understand, and apply it [21], thereby contributing to improved health outcomes.

## Methods

### Study Design and Setting

A cross-sectional 10-minute survey was conducted among the UK adult population. The web-based survey was administered through YouGov from November 22 to December 4, 2024. This study was a secondary analysis of the dataset collected. The study used the STROBE (Strengthening the Reporting of Observational Studies in Epidemiology) guidelines for cross-sectional studies [37] (Checklist 1).

### Participants

Upon signing up to YouGov, prospective respondents agreed to their terms and conditions and acknowledged the privacy notice [38,39]. YouGov panel members received an email invitation that introduced the study and included a link to the web-based survey. Participants were required to be internet users, aged 18 years or older, live in the United Kingdom, and able to complete the survey in English. Quotas were used to aim for representativeness with regard to age, sex, region, ethnicity, and National Readership Survey (NRS) social grade [40]. Participants received 50 points on the YouGov platform to compensate them for their time, the equivalent of £0.50 (approximately US $0.67).

### Sample Size

A minimum sample size was calculated using the formula:


$$n=\frac{Z^{2}\;p(1-p)}{\varepsilon^{2}}$$

where *n* is the required sample size, *Z* is the *Z* score, *p* is the estimated population proportion, and *ε* is the margin of error. Assuming a 95% CI (*Z*=1.96), a conservative estimated proportion (*p*=0.5), and a margin of error of 5% (*ε*=0.05), the minimum required sample size was calculated to be 385. Given the large population size of 52.8 million people aged 20 years and older in the United Kingdom [41], a finite population correction was not applied as it would have a negligible effect on the estimate. A final sample of 1525 participants was included in the analysis.

### Survey Development

The survey was developed by a multidisciplinary team comprising medical doctors, nurses, and health services researchers. The questionnaire operationalized the PROGRESS-Plus framework, which illustrates the multiple factors that can contribute to health inequalities in a population [42,43]. It collected information on participants’ sociodemographic, economic, and social characteristics, as well as data about health and DHL (Multimedia Appendix 1).

### Ethical Considerations

Consent to enter the study was sought from each participant after a full explanation was given and before accessing the survey. All participants were free to withdraw at any time by closing the browser window where they were completing the survey; there were no consequences for withdrawal. Study participants and YouGov gave permission for these data to be used for research purposes. Approval for this study was obtained from the Head of Department and the Research Governance and Integrity Team (reference 7750506); the study was also registered and approved via Imperial’s Data Asset Registration Tool. The study followed the ethical principles of the World Medical Association outlined in the Declaration of Helsinki and subsequent amendments [44].

### Variables

The predictor variables analyzed were UK region, urbanicity, ethnicity, primary language spoken within the household, employment status, sex, religion, educational attainment, annual household income, NRS social grade [40], frequency of meeting friends and family, age group, presence of health conditions, and limitations to daily activities. These characteristics were mapped against the PROGRESS-Plus framework [42], as shown in Multimedia Appendix 1. Urbanicity was measured using self-report; participants were asked “Do you live in an urban, suburban, or rural area?” with answer options urban, suburban, rural. At the analysis stage, this variable was dichotomized to give urban and nonurban as groups. NRS social grade is a classification system based on the occupation of the household’s chief income earner, their qualifications, and the number of people they are responsible for [40]. This yields 6 categories, A, B, C1, C2, D, and E, with A representing higher managerial roles and E representing casual or lowest grade workers, or those who are unemployed and on state benefits or pensions [40]. These groups were recategorized to give ABC1 and C2DE, where the latter is the lower social grade.

The outcome variable was DHL, as measured by eHEALS [28]. This is a simple, 8-item tool, including items such as “I know how to use the Internet to answer my health questions” and “I can tell high quality from low quality health resources on the Internet” [28] (Multimedia Appendix 2). Each item uses a 5-point Likert scale with response options ranging from “strongly disagree” to “strongly agree” [28]. Overall eHEALS score was calculated by summing answers across the 8 items for each participant, giving a final score from 8 to 40 [28]. In this study, eHEALS was dichotomized to present high and low DHL, with a score <26 representing low DHL [34,45-47]. This cutoff was first applied by Richtering et al [45] and has since been adopted across multiple studies, facilitating comparison with the existing literature [34,46,47]. The internal consistency of eHEALS data in this sample was calculated using Cronbach α.

### Statistical Analysis

Descriptive statistics were performed using total and relative frequencies for categorical variables. Participants categorized as having low and high DHL were compared using bivariate analysis. Prior to analysis, linear regression using the continuous eHEALS score was explored; however, model diagnostics indicated that key assumptions were violated, including constant variance and normality of residuals, likely reflecting the nonnormal distribution of eHEALS score in this sample. Logistic regression using a dichotomized eHEALS score was therefore adopted as the primary analytical approach. Predictor reference categories were selected based on theoretical relevance and interpretability. A multivariable logistic regression model was built using the enter method where all possible predictors were included as independent variables, irrespective of their significance at the univariable analysis stage. Before building the multivariable model, associations between predictor variables were assessed to check for multicollinearity. Chi-square tests (or Fisher exact tests where expected cell count was <5) were used for this purpose; predictors were omitted from the multivariable model where the association between them was considered problematic, defined as *P*<.05 and Cramer V≥0.3. After investigation of associations between predictor variables, region, employment status, primary language, income, and health condition were omitted from the multivariable model due to problematic associations with other predictors.

Descriptive statistics and prevalence estimates were calculated using the full sample. Most variables had low levels of missingness (0%‐5%), with the exception of income; income was excluded from the multivariable model on this basis, alongside multicollinearity with other variables. Little’s missing completely at random (MCAR) test [48] indicated that data were not MCAR. Further analysis revealed that missingness in certain sociodemographic variables, namely, education and limited daily activities, was associated with significantly lower DHL scores, suggesting a systematic relationship between missingness and the outcome. Multiple imputation by chained equations (MICE) was therefore used as the primary approach to handle missing data, using the mice package in R (R Core Team) [49-51]. Twenty imputed datasets were generated, reflecting the proportion of incomplete cases in the analytical sample (198/1525, 13%) [52]. Appropriate imputation methods were specified for each variable type: logistic regression for binary variables (namely, urbanicity, religion, and limited daily activities) and polytomous logistic regression for unordered categorical variables with more than 2 levels (namely, education). All covariates from the multivariable model and outcome variable of DHL were included in the imputation model, alongside auxiliary variables (region, employment status, primary language, income, and health condition) to improve imputation quality. Convergence was assessed visually using trace plots, and the distribution of imputed values was compared with observed distributions to verify plausibility. The logistic regression model was fitted within each imputed dataset, and results were pooled using Rubin’s rules [49,50].

The estimated effects on DHL were expressed as odds ratios (ORs), alongside 95% CIs for each variable; these values were adjusted for all other variables in the multivariable model. *P* values were also reported, with *P* values <.05 considered statistically significant. All analysis was performed using R software (version 4.4.2; R Core Team) [53].

### Sensitivity Analyses

Given the unclear and contradictory relationship between health conditions and DHL reported in the literature [17,31,32], a subgroup analysis was conducted among participants who reported any health condition to explore whether health condition status modified the relationship between sociodemographic, economic, and social factors and DHL. Univariable and multivariable logistic regression was repeated within this subgroup, using the same predictor variables as the primary analysis, with health condition excluded as a predictor given that all participants in this subgroup reported at least 1 condition.

To assess the robustness of findings to the eHEALS cutoff, a sensitivity analysis was conducted using the sample median, 31, as an alternative threshold. To explore potential effect modification, interaction terms for theoretically motivated pairs of predictors (age by sex, age by education, and sex by education) were tested alongside the primary model. To assess the potential impact of missing data on regression estimates, complete case analysis (CCA) was conducted as a sensitivity analysis alongside the primary multiple imputation analysis, resulting in the exclusion of 198 (198/1525, 13%) participants from the multivariable analysis. McFadden adjusted *R*^2^, the likelihood ratio test, and Akaike Information Criterion (AIC) were used to assess explanatory power and fit for the CCA model. As these statistics are not straightforwardly pooled after MICE [51], and no fully standardized approach currently exists, they are reported for the CCA only.

## Results

### Descriptive Characterization of Participants

**Figure 1.**
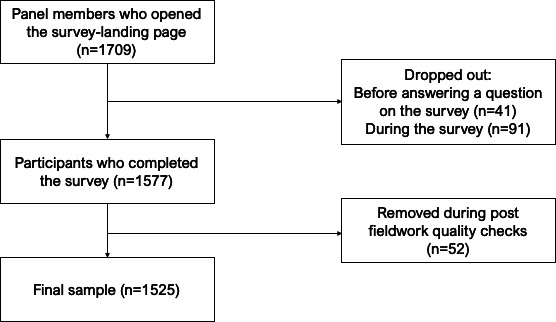
Study sample flow diagram.

The final sample analyzed consisted of 1525 participants, giving a completion rate of 89.2%, as illustrated in Figure 1. Due to the survey approach, it was not possible to determine reasons for dropout. The majority of respondents lived in England (n=1281, 84%), in urban areas (n=1144, 75%), and were of White ethnicity (n=1349, 88.5%) (Table 1). A quarter of respondents were aged 65 years or older (n=371, 24.3%) and just less than half were male (n=734, 48.1%) (Table 1). Over half (n=881, 57.8%) of the sample were in employment, while 24.3% (n=370) were retired. Half reported below degree level education (n=747, 49.0%) (Table 1).

**Table 1.** Participants’ characteristics, including DHL^a^, and sociodemographic, economic, social, and health characteristics, total and split by DHL level.

Variable	Total (N=1525)	Low DHL (n=320)	High DHL (n=1205)	*P* value^b^
eHEALS^c^, median (IQR)	31 (27‐32)	22 (19‐24)	32 (30‐33)	<.001
UK region, n (%)				.44
England	1281 (84)	261 (81.56)	1020 (84.65)	
Wales	81 (5.31)	17 (5.31)	64 (5.31)	
Scotland	135 (8.85)	34 (10.63)	101 (8.38)	
Northern Ireland	28 (1.84)	8 (2.50)	20 (1.66)	
Missing	0 (0)	0 (0)	0 (0)	
Urbanicity, n (%) ^d^				.21
Urban	1144 (75.02)	232 (72.50)	912 (75.68)	
Nonurban	316 (20.72)	69 (21.56)	247 (20.50)	
Missing	65 (4.26)	19 (5.94)	46 (3.82)	
Ethnicity, n (%)				.50
White	1349 (88.46)	287 (89.69)	1062 (88.13)	
Other	176 (11.54)	33 (10.31)	143 (11.87)	
Missing	0 (0)	0 (0)	0 (0)	
Primary language, n (%)				>.99
English	1464 (96)	307 (95.94)	1157 (96.02)	
Other	61 (4)	13 (4.06)	48 (3.98)	
Missing	0 (0)	0 (0)	0 (0)	
Employment status, n (%)				.48
Working	881 (57.77)	176 (55)	705 (58.51)	
Student	43 (2.82)	10 (3.13)	33 (2.74)	
Retired	370 (24.26)	87 (27.19)	283 (23.49)	
Unemployed/ not working	154 (10.10)	28 (8.75)	126 (10.46)	
Other	77 (5.05)	19 (5.94)	58 (4.81)	
Missing	0 (0)	0 (0)	0 (0)	
Sex, n (%)				<.001
Male	734 (48.13)	187 (58.44)	547 (45.39)	
Female	791 (51.87)	133 (41.56)	658 (54.61)	
Missing	0 (0)	0 (0)	0 (0)	
Religious, n (%)				.33
Yes	677 (44.39)	132 (41.25)	545 (45.22)	
No	778 (51.02)	170 (53.13)	608 (50.46)	
Prefer not to say	70 (4.59)	18 (5.63)	52 (4.32)	
Missing	0 (0)	0 (0)	0 (0)	
Educational attainment, n (%)				<.001
Below degree level	747 (48.98)	193 (60.31)	554 (45.98)	
Undergraduate degree	322 (21.11)	53 (16.56)	337 (27.97)	
Postgraduate degree or higher	390 (25.57)	50 (15.63)	272 (22.57)	
Don’t know or prefer not to say	66 (4.33)	24 (7.50)	42 (3.49)	
Missing	0 (0)	0 (0)	0 (0)	
Social grade, n (%)				<.001
ABC1	809 (53.05)	138 (43.13)	671 (55.68)	
C2DE	716 (46.95)	182 (56.88)	534 (44.32)	
Missing	0 (0)	0 (0)	0 (0)	
Annual household income, n (%)				.001
Less than £20,000	295 (19.34)	70 (21.88)	225 (18.67)	
£20,000–£39,999	391 (25.64)	75 (23.44)	316 (26.22)	
£40,000–£59,999	241 (15.80)	40 (12.50)	201 (16.68)	
£60,000 or greater	277 (18.16)	45 (14.06)	232 (19.25)	
Prefer not to say	321 (21.05)	90 (28.13)	231 (19.17)	
Missing	0 (0)	0 (0)	0 (0)	
Frequency of meeting with family or friends, n (%)				.02
Never or rarely	177 (11.61)	50 (15.63)	127 (10.54)	
Weekly or monthly	970 (63.61)	203 (63.44)	767 (63.65)	
Daily	378 (24.79)	67 (20.94)	311 (25.81)	
Missing	0 (0)	0 (0)	0 (0)	
Age group (years), n (%)				.02
18‐44	645 (42.30)	115 (35.94)	530 (43.98)	
45‐64	509 (33.38)	111 (34.69)	398 (33.03)	
≥65	371 (24.33)	94 (29.38)	277 (22.99)	
Missing	0 (0)	0 (0)	0 (0)	
Health condition, n (%)				.005
Yes	850 (55.74)	168 (52.50)	682 (56.60)	
No	599 (39.28)	125 (39.06)	474 (39.33)	
Prefer not to say	76 (4.98)	27 (8.44)	49 (4.07)	
Missing	0 (0)	0 (0)	0 (0)	
Limited activity, n (%)				<.001
Yes	432 (28.33)	89 (27.81)	343 (28.46)	
No	1058 (69.38)	212 (66.25)	846 (70.21)	
Missing	35 (2.30)	19 (5.94)	16 (1.33)	

a DHL: digital health literacy.

b *P* values reported in this table correspond to statistical tests of differences between low and high DHL groups. For continuous variables, *P* values were derived from Welch 2-sample *t* tests; for categorical variables, *P* values were derived from chi-square tests (or Fisher exact tests where expected cell count was <5).

c eHEALS: eHealth Literacy Scale.

d Participants were asked “Do you live in an urban, suburban, or rural area?” with answer options urban, suburban, and rural; this variable was dichotomized to give urban and nonurban as groups.

### Assessment of DHL in the United Kingdom

Analysis indicated that eHEALS scores were not normally distributed (Multimedia Appendix 3), as confirmed by the Shapiro-Wilk test (*P*<.001). Median eHEALS was 31, with an IQR of 27‐32. The internal consistency of the data collected using eHEALS in this study was high (Cronbach α=0.93) and comparable with reliability estimates reported in previous studies [21,27,28]. A fifth (320/1525, 21%) of the sample had a low level of DHL, based on the 26-point cutoff. Comparing the subpopulations with low and high levels of DHL, those with low DHL were more likely to be male, belong to an older age group, have a lower level of education, belong to a lower NRS social grade, and have a lower household income (Table 1). The eHEALS score IQR was wider among those with lower DHL, further indicating inequality in terms of the distribution of DHL in the UK population.

**Figure 2.**
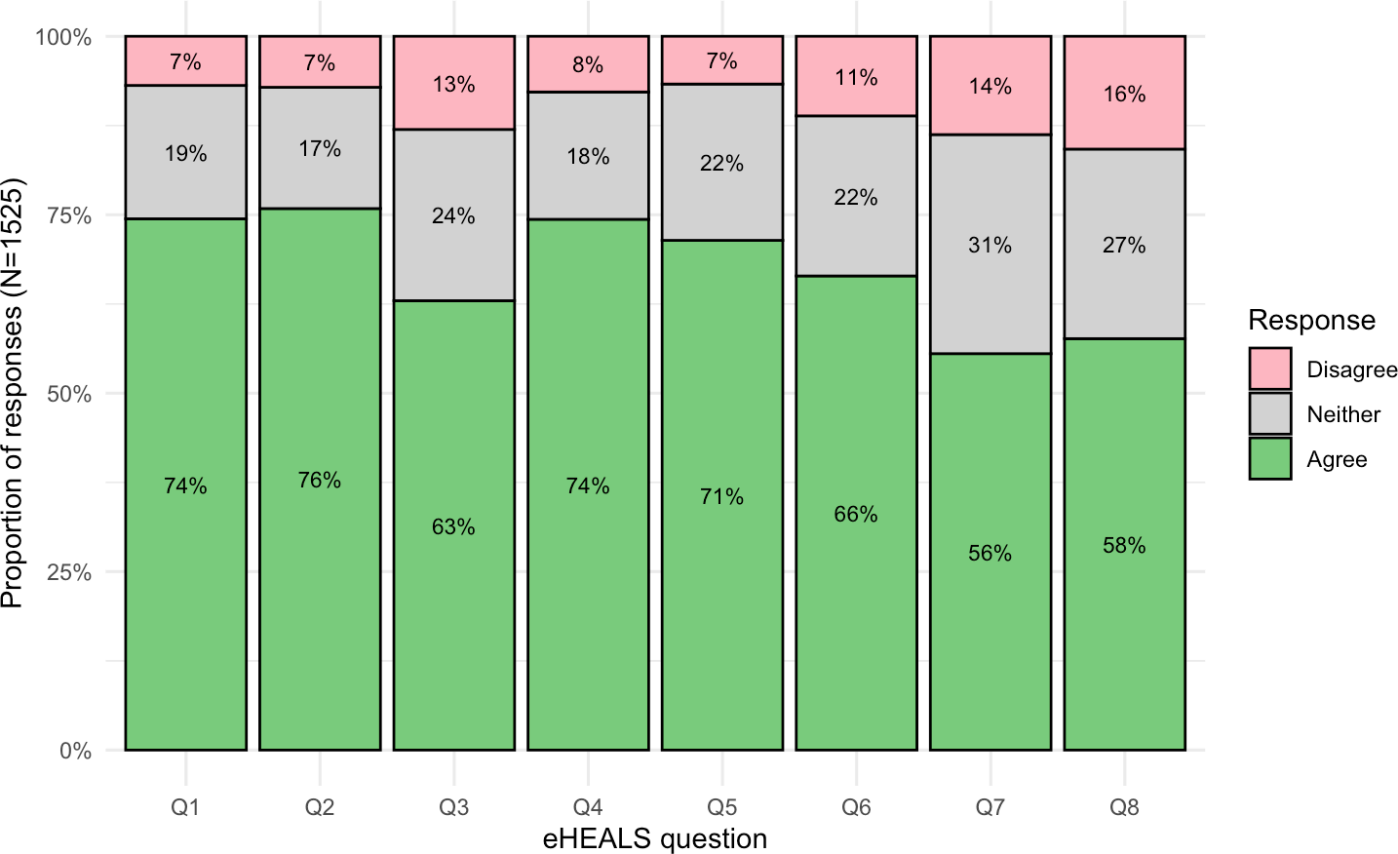
Participant responses to eHEALS items (N=1525). Response categories were grouped into agree (including “strongly agree” and “agree”), disagree (including “strongly disagree” and “disagree”), or neither. Q1: I know how to find helpful health resources on the internet; Q2: I know how to use the internet to answer my health questions; Q3: I know what health resources are available on the internet; Q4: I know where to find helpful health resources on the internet; Q5: I know how to use the health information I find on the internet to help me; Q6: I have the skills I need to evaluate the health resources I find on the internet; Q7: I can tell high quality from low quality health resources on the internet; and Q8: I feel confident in using information from the internet to make health decisions. eHEALS: eHealth Literacy Scale.

As shown in Figure 2, participants had the lowest level of agreement with “I feel confident in using information from the Internet to make health decisions” (Q8; 879/1525, 57.6%) and *“*I can tell high quality from low quality health resources on the Internet” (Q7; 847/1525, 55.5%).

### Factors Associated With DHL

**Figure 3.**
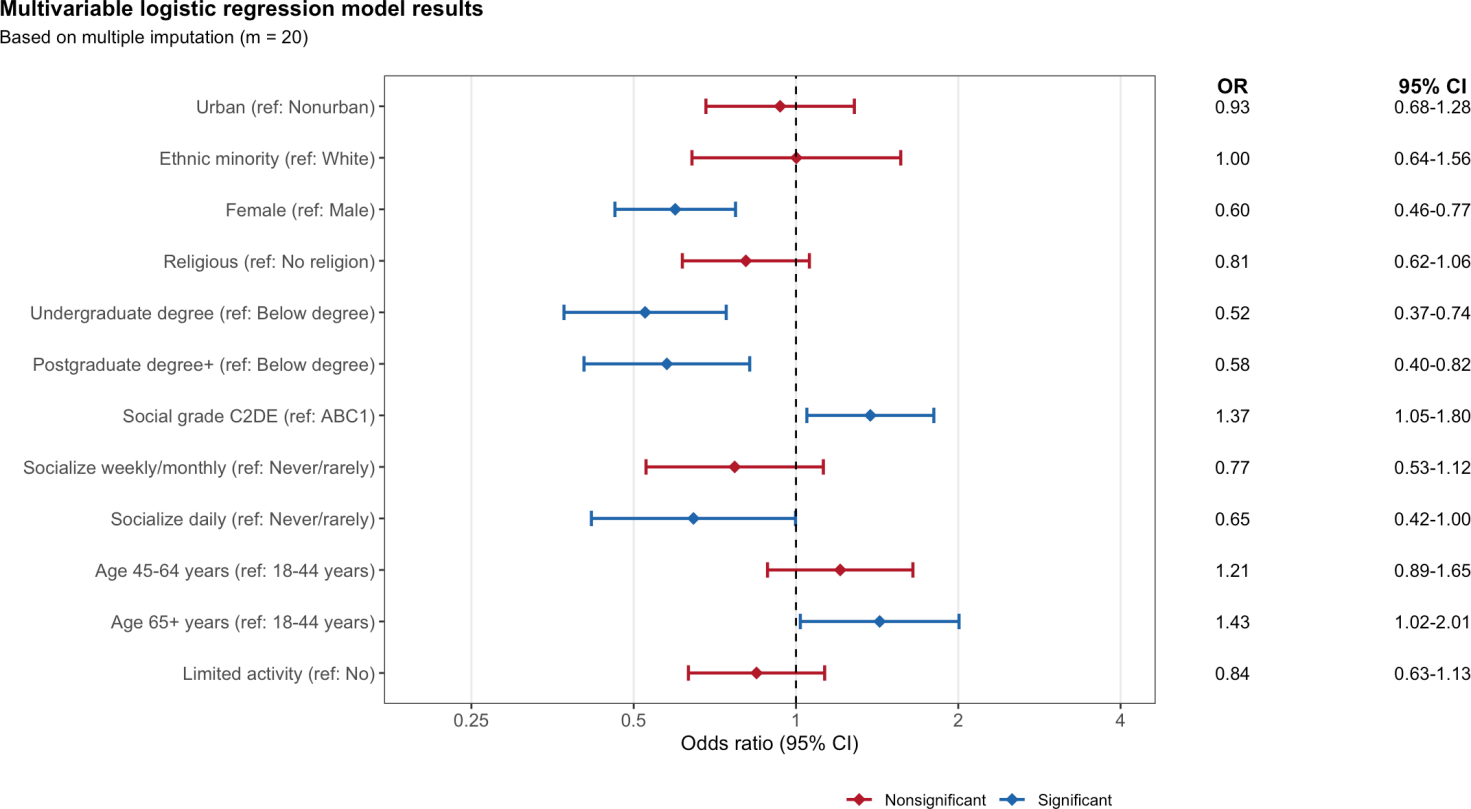
Forest plot displaying sociodemographic, economic, and social associates of digital health literacy. Adjusted ORs and 95% CIs are shown. OR: odds ratio.

Univariable regression results are presented in Multimedia Appendix 4. In the multivariable model based on the imputed dataset, age, social grade, sex, education, and frequency of meeting with family or friends were significant associates of DHL (Figure 3; Multimedia Appendix 4). Those aged 65 years and older, compared with those in the age group of 18‐44 years, had significantly higher odds of low DHL (OR 1.43, 95% CI 1.02‐2.01; *P*=.04). Those belonging to the lower NRS social grade, C2DE, also had significantly higher odds of low DHL than those belonging to the higher social grade (OR 1.37, 95% CI 1.05‐1.80; *P*=.02). Those of female sex had lower odds of low DHL (OR 0.60, 95% CI 0.46‐0.77; *P*<.001), as did those with an undergraduate or postgraduate degree or higher, compared with those educated to below degree level (undergraduate degree OR 0.52, 95% CI 0.37‐0.74, *P*<.001; postgraduate degree or higher OR 0.58, 95% CI 0.40‐0.82, *P*=.002). Participants who reported meeting with family or friends daily, compared with those who reported they did this either never or rarely, had marginally significantly lower odds of low DHL (OR 0.64, 95% CI 0.42‐1.00; *P*=.05).

### Sensitivity Analyses

Among those participants with a health condition, female sex, being educated to undergraduate degree or postgraduate degree or greater, and meeting with family or friends daily continued to be statistically significant associates of DHL (Multimedia Appendix 5). In this subgroup of participants, older age and lower social grade were no longer significantly associated with low DHL (age of 65 years and older OR 1.18, 95% CI 0.74‐1.88, *P*=.48; C2DE OR 1.41, 95% CI 0.97‐2.04, *P*=.07) (Multimedia Appendix 5).

Using the median eHEALS score, 31, as an alternative cutoff produced results consistent with the primary analysis, with age of 65 years and older, social grade, sex, education, and frequency of meeting with family or friends remaining statistically significant associates of low DHL (Multimedia Appendix 6). The direction of effect was consistent across both models, although the magnitude of associations differed slightly. Notably, the age group of 45‐64 years reached statistical significance in the median cutoff model (OR 1.38, 95% CI 1.07‐1.76; *P*=.01) (Multimedia Appendix 6), suggesting that this is a borderline finding sensitive to the choice of threshold. Overall, these findings support the robustness of the primary findings.

Interaction terms were tested for 3 theoretically motivated pairs of predictors: age by sex, age by education, and sex by education (Multimedia Appendix 7). All age by education and sex by education interaction terms were nonsignificant. One age by sex interaction term (female × age 45‐64 years) reached statistical significance (*P*=.009); however, the overall improvement in model fit was negligible [54], with an AIC difference of 2 points between the interaction model and the primary model, based on a CCA approach. The primary model without interaction terms was therefore retained as the more parsimonious specification.

Results of the CCA are presented in Multimedia Appendix 8 to allow direct comparison of ORs and standard errors. The direction of effect was consistent across all variables between the 2 models. Coefficient estimates for the strongest sociodemographic, economic, and social associates of low DHL, including female sex, educational attainment, and older age, were attenuated in the imputed model compared with the CCA model, consistent with the systematic association between missingness and lower DHL scores identified in our missing data analysis. However, estimates for social grade C2DE and frequency of socializing were slightly larger in the imputed model, suggesting that the impact of missing data handling was variable across predictors. Notably, estimates for religion and age group of 45‐64 years were significant in the complete case model but not in the imputed model, while those for social grade C2DE and daily socializing reached significance in the imputed model but not in the complete case model. McFadden adjusted *R*^2^ for the CCA model was 0.06, indicating modest explanatory power of the included predictors. A likelihood ratio test comparing the null model with the fitted CCA model indicated a statistically significant improvement in model fit (*χ*^2^_12_=80.15; *P*<.001), suggesting that the predictors significantly improved the model’s ability to explain variation in DHL level. The AIC was 1258.8.

## Discussion

### Principal Findings

The median eHEALS score in this population was 31, which is defined as a high level of DHL. However, a fifth (320/1525, 21%) of the participants had a low level of DHL. Participant agreement was lowest regarding using internet-based information to make health decisions and distinguishing high- and low-quality health resources on the internet. Older participants, specifically those aged 65 years and older, and those in the lower social grade (C2DE) were more likely to have low DHL after adjustment. Conversely, female participants, those with greater educational attainment, and those who socialized with family and friends more frequently were less likely to have low DHL. No evidence of a significant association was found between DHL and urbanicity, ethnicity, religion, or limited activity.

### Comparison With Existing Literature

The median eHEALS score in this study was slightly higher relative to previous studies conducted in the United Kingdom [32,33,55]. This study identified a lower proportion of participants with low DHL (320/1525, 21%) compared with previous studies, although this difference could be attributed to between-country differences in DHL [32].

Despite the frequent use of eHEALS to measure DHL, relatively few studies report levels of agreement with individual scale items. However, among those studies that do report item-level breakdowns, there is consistency in which items receive the highest level of disagreement [27,56,57]. These findings indicate that populations struggle when it comes to critically engaging with, assessing, and applying digital health information.

Regarding the significant associates of DHL, a summary of findings compared with the previous literature is presented in Table 2. The observed relationship between education and DHL in this study aligns with evidence highlighting education as one of the most consistent associates of eHEALS scores [10,33,56].

**Table 2.** Summary of findings, compared with the previous literature and mapped to the PROGRESS-Plus framework.

Predictor variable	Summary of findings	Comparison with the literature
Region	This study found no effect of UK region on DHL^a^ in the unadjusted analysis.	To date, no studies have investigated the effect of UK region on DHL.
Urbanicity	This study found no effect of urbanicity on DHL in either unadjusted or adjusted analysis.	This is in line with a previous UK-based study [33], although this remains an understudied variable with regard to DHL.
Ethnicity	This study found no effect of ethnicity on DHL in either unadjusted or adjusted analysis.	This supports previous evidence from 2 UK-based studies and an international review of the literature [10,32,33].
Primary language	This study found no effect of primary language spoken on DHL in unadjusted analysis.	This supports findings from a US-based study, which included participants who primarily spoke Spanish at home [58], but negates findings from Sweden, where Arabic-speaking participants were more likely to have low DHL [34].
Employment status	This study found no effect of employment status on DHL in unadjusted analysis.	This broadly supports the evidence base [10]. On the other hand, a study in China found that nonemployed participants had lower eHEALS^b^ scores than their employed counterparts [35].
Sex	Sex was associated with DHL in both unadjusted and adjusted analyses.	This contrasts with some previous literature, which reports mixed effects [10,32].
Religion	This study found no effect of religious self-identification on DHL in unadjusted or adjusted analysis.	To date, no studies have investigated the effect of religion on DHL.
Education	Education was associated with DHL in both unadjusted and adjusted analyses.	This supports previous evidence, including an international review of the literature [10,33].
Socioeconomic status	NRS^c^ social grade was associated with DHL in both unadjusted and adjusted analyses. It was not associated with DHL among those participants with a chronic health condition. An annual income of £60,000 or greater was associated with DHL in unadjusted analysis.	Socioeconomic status is measured in many ways in the literature, making comparison challenging. When looking at income specifically, this has been found to be a determinant of eHEALS score [10,31].
Social support	Frequency of meetings with family or friends was associated with DHL in both unadjusted and adjusted analysis. This association was retained across sensitivity analysis using the median eHEALS value as a cutoff and among participants with a health condition. The association was nonsignificant in the CCA^d^.	Social support has been suggested as a determinant of DHL in a review [10]. Although this is operationalized in different ways across studies, there is some indication that social support could be a protective factor to DHL [35,59].
Age (years)	Older age (65 years and older) was associated with DHL, in both unadjusted and adjusted analyses. It was not associated with DHL among those participants with a chronic health condition. Middle age (45‐64 years) is not a significant associate of low DHL in the primary analysis, although it reached significance in the sensitivity analysis that used the median eHEALS value as a cutoff. The interaction term female × 45‐64 years reached statistical significance.	This broadly supports previous evidence from 2 UK-based studies and 2 separate meta-analyses [10,31-33]. The lack of effect among participants with a chronic health condition also supports previous findings [56].
Chronic health conditions	This study found no effect of the presence of a health condition on DHL in unadjusted analysis.	This contrasts with findings from an international meta-analysis, which found that participants with existing disease or at a higher risk for disease had lower eHEALS scores [31].
Limited activity	This study found no effect of limited activity on DHL in unadjusted or adjusted analysis.	To date, no studies have investigated the effect of limited activity on DHL.

a DHL: digital health literacy.

b eHEALS: eHealth Literacy Scale.

c NRS: National Readership Survey.

d CCA: complete case analysis.

The finding that age is a statistically significant sociodemographic associate of DHL is also consistent with prior research [10,31-33]. Qualitative research suggests that older adults may experience barriers relating to technological discomfort; lack of perceived advantage; concerns about privacy, security, and access; and lack of self-alignment with the imagined user [60]. The association between age group of 65 years and older and low DHL was robust across all analyses, consistent with this body of literature. In contrast, the association between age group of 45‐64 years and low DHL was less consistent, showing sensitivity to the choice of eHEALS cutoff and a significant interaction term with female sex, despite the interaction model not offering a meaningful improvement in overall model fit. This pattern may suggest that the relationship between middle age and DHL is heterogeneous, potentially varying by sex, and warrants further investigation in future studies.

Notably, among participants with chronic health conditions, age was no longer a statistically significant sociodemographic associate of DHL across any age group. One possible explanation is that individuals living with health conditions may engage with online health information out of necessity regardless of age, as the personal relevance of seeking and applying health information is heightened by their health status. This is consistent with findings from Stellefson et al [56], who reported that age was not a predictor of DHL among US patients with chronic obstructive pulmonary disease after adjusting for multiple covariates [56]. Taken together, these findings point to important context-specific nuances whereby health necessity may override typical age-related patterns.

This study identified sex as a statistically significant sociodemographic associate of DHL, even after adjustment for covariates, and across sensitivity analyses. This finding contrasts with some previous research [32]. However, other studies suggest a more complex picture, likely reflecting heterogeneity in both study populations and health system contexts [10]. It is worth noting that eHEALS measures perceived confidence in using digital health information rather than objectively assessed skill, and this distinction may be relevant to interpreting the observed sex difference. Research has suggested that eHEALS assesses self-efficacy rather than real-world performance [61], and a study comparing eHEALS scores with actual task performance concluded that its emphasis on self-perception means that it does not accurately predict performance [21]. A cross-sectional study of UK university students found that eHEALS was significantly positively correlated with general self-efficacy [55], further supporting this interpretation. Men and women may differ in health information-seeking behavior and self-efficacy reporting styles independently of actual digital competence, which could contribute to the observed sex difference. The broader behavioral evidence supports this interpretation; women have been shown to engage more frequently with digital health tools, with 64.4% of online consultations in Great Britain completed by female patients [62], and a higher proportion of women than men reporting use of digital technology to support their health in a nationally representative Welsh survey (71% vs 65%) [63]. This suggests that the sex difference observed in this study may partly reflect differential engagement with and confidence in digital health resources, rather than a straightforward difference in underlying digital skill.

In this study, urbanicity and ethnicity had no effect on DHL. This is consistent with previous studies, which also reported no significant relationship between these variables and eHEALS score [10,32,33]. However, these findings should be interpreted with caution. The relatively low proportion of non-White participants may have limited the statistical power to detect an effect. Similarly, primary language had no effect on DHL, in contrast to findings among Arabic- and Swedish speakers in Sweden [34]. However, the predominance of English as the primary language in the sample likely limited the ability to detect an effect. The frequency of meeting with family or friends was a statistically significant sociodemographic associate of DHL. Wider evidence regarding the role that social support plays in DHL remains inconclusive [10,35,56].

### Strengths, Limitations, and Future Research

To our knowledge, this is the first study to comprehensively examine the sociodemographic, economic, and social factors associated with DHL in the United Kingdom. This study adopts an equity-oriented approach by incorporating a broad range of factors, including social support and aspects of cultural capital. The study benefits from a large sample that is broadly representative of the UK adult population. eHEALS is a widely used instrument that has been validated in previous research.

The principal limitation of this study is related to the purely online dissemination of the survey that inadvertently excluded individuals who do not use the internet. According to Ofcom in 2024 [64], 5% of UK adults reported never accessing the internet either at home or elsewhere, and the same percentage reported having no internet access at home. While this represents a relatively small proportion of the population, those excluded by virtue of being offline are systematically more likely to have low DHL. Consequently, the reported prevalence of low DHL (21%) should be interpreted as a likely underestimate of the true prevalence in the general UK population, and findings are most applicable to UK internet users rather than the population as a whole. Additionally, the study was unlikely to capture the most socially excluded populations, who are known to face pronounced digital inequities [65]. To mitigate the risk of bias, few exclusion criteria were employed. Future research should consider mixed-mode recruitment and survey dissemination strategies to reach those without internet access [66], in order to better characterize DHL in the full UK population.

Furthermore, the study sample was not fully representative in terms of ethnicity or language. While 88% (1329/1525) of participants identified as White, this compares with 82% in the 2021 England and Wales Census [67]. Because of small subgroup sizes, all non-White ethnic groups were grouped together, limiting the ability to examine between-group differences. Similarly, 96% (1464/1525) of the sample reported English as their primary language, compared with 91% in the Census [68]. This could be due to the survey requiring participants to be English-speaking; future research could overcome this by translating the survey tool into a range of languages.

DHL was self-reported using eHEALS, which may not accurately reflect participants’ actual abilities [14,21]. In addition, eHEALS was created almost 20 years ago, and it may not fully capture newer forms of digital health technology [10,69]. Despite this, eHEALS remains the most frequently used tool to measure DHL [10,14,30]. Future studies should aim to use an updated validated instrument [70]. For instance, the HLS_19_-DIGI instrument considers a broader range of digital information sources and has recently been validated in 13 European countries [71]. Performance-based tests could also be used as an objective assessment of DHL [70,72].

A further limitation relates to missing data. Little’s MCAR test [48] indicated that data were not MCAR, and missingness in education and disability status was associated with significantly lower DHL scores, suggesting a systematic relationship between missingness and the outcome. Multiple imputation was therefore used as the primary analytical approach, with CCA presented as a sensitivity analysis. While the direction of effect was consistent across both models, some differences in the significance of associations were observed. These differences highlight the sensitivity of borderline findings to the method of missing data handling, and results for religion and age group of 45‐64 years, as well as social grade and frequency of socializing, should be interpreted with caution. Although MICE is considered the preferred approach when the MCAR assumption is not met, it relies on the assumption that data are missing at random, which cannot be formally verified [50,73]; the possibility of data missing not at random cannot be excluded [50,73]. As descriptive analyses and prevalence estimates were conducted on the full sample, the reported prevalence of low DHL (21%) is not affected by missing data handling.

The dichotomization of eHEALS scores, while consistent with the existing literature, carries inherent limitations including potential loss of statistical power and the imposition of an artificial binary distinction. The cutoff of 26 was adopted to facilitate comparison with prior research, although its derivation is not fully documented in the original source [45]. Sensitivity analysis using the median as an alternative cutoff supported the robustness of the primary findings with the direction of effect consistent across both models. However, the association between age group of 45‐64 years and low DHL was sensitive to the choice of threshold, reaching significance only in the median cutoff model, and should therefore be interpreted with caution.

The study’s cross-sectional design limits any ability to draw causal inferences. Finally, although the regression model was statistically significant, it accounted for only 6% of the variability in DHL in the complete case model, leaving the majority of variance unexplained. As model fit statistics are not straightforwardly pooled after multiple imputation [51,74], this figure is reported for the complete case model only; however, the imputed model was similarly consistent with modest explanatory power. While the identified associations are reliable, the sociodemographic, economic, and social factors examined are not strong associates of DHL at the individual level, and the model should not be interpreted as a predictive tool. This is consistent with the broader literature, which suggests that DHL is a complex, multidimensional construct shaped by factors beyond sociodemographic characteristics alone. Unmeasured variables that could account for the remaining variance include access to mobile devices [33], frequency of use of digital technologies [31,75], self-efficacy in using such tools [55,76], perceived importance, and perceived usefulness [31]. Future research should seek to incorporate these dimensions to develop a more comprehensive explanatory model of DHL. A further dimension not captured in this study is trust in digital health information sources. Trust in digital health technologies has been shown to be influenced by impediments, such as limited accessibility, fear of data exploitation, and poor information quality, as well as enablers including ease of use, customizable design features, and privacy [77]. Trust has been recognized as an important influence on digital health care use, adoption, acceptance, and usefulness [78]. A full exploration of trust as a factor associated with DHL is beyond the scope of this study but represents an important avenue for future research.

### Implications for Practice and Policy

Evidence from this study and prior research suggests that DHL is unevenly distributed across the UK population. Therefore, rather than adopting a one-size-fits-all approach, the NHS, public health bodies, and local authorities should tailor communication, services, and interventions based on the DHL needs of different population groups.

Two complementary strategies can be implemented. First, individual DHL can be improved through educational programs, tailored to account for variation in DHL by age, education, socioeconomic status, and sex. Based on this study, content should focus on applying internet-based information to make health decisions and discerning health resource quality. In terms of delivery format, demonstrations, in-person workshops, and tutorials have the potential to motivate individuals to engage with digital technologies [14]. Particularly among older adults, ongoing and iterative support is likely to be more effective than one-time instruction [79]. Given the potential role of social support in determining DHL, interventions that involve community members or peers who have higher levels of DHL may have greater success [14]. This study suggests that necessity-driven engagement among people with health conditions may attenuate age- and socioeconomic-related barriers. Therefore, chronic condition management pathways represent a potential entry point for DHL support. Integrating a brief DHL screening tool into routine intake processes for patients with chronic conditions, flagging those scoring below a threshold to receive tailored DHL support, could provide a scalable and targeted approach to identifying and supporting individuals with low DHL at a point of existing engagement with health services [80].

Second, digital health solutions should be developed with user-centered, inclusive design principles. Solutions should be tailored toward anticipated users’ DHL levels, ideally using co-design processes that involve users with lower DHL [32]. Digital health tools should prioritize content that is applicable to users’ day-to-day life, for example, by helping to inform health decisions. For older adults, adjusting the size of display icons, tailoring volume settings, and simplifying interfaces of digital tools and apps may improve usability [32].

Finally, to best serve the needs of all population groups, regular and localized monitoring of DHL is essential. Identifying populations with lower levels of DHL would enable more responsive health planning and guide resource allocation, creating a more equitable health system.

### Conclusions

This study found that 21% of the UK population had a low level of DHL and revealed sex, educational attainment, socioeconomic status, age, and frequency of socializing as statistically significant associates of DHL. Those aged 65 years and older were more likely to have low DHL than younger participants when adjusting for other factors. Those in the lower social grade (C2DE) were more likely to have low DHL than those in the higher social grade. Those of female sex were less likely to have low DHL, as were those with an undergraduate or postgraduate degree or higher, compared with those educated to below degree level. Those who socialized with friends and family daily were less likely to have low DHL. The modest explanatory power of the model suggests that additional factors beyond those examined play an important role, and the identified associates should not be interpreted as strong individual-level predictors of DHL. Tailored educational interventions and user-centered digital health solutions can help address the uneven distribution of DHL across the UK population. Notably, subgroup analysis among those with chronic health conditions suggests that necessity-driven engagement with health information may attenuate age- and socioeconomic-related barriers among those living with chronic conditions. Intervention strategies should therefore be tailored to health status, with chronic condition management pathways representing a valuable opportunity to support DHL across age groups. The findings of this study echo concerns in the literature around the limited applicability of eHEALS in the current digital health environment [21,69,70,81]. The development of an updated measurement tool could support the identification of population subgroups with low DHL.

## Supplementary material

10.2196/89136 Multimedia Appendix 1 Operationalization of PROGRESS-Plus framework in the survey.

10.2196/89136 Multimedia Appendix 2 eHealth Literacy Scale. Each item is answered on a 5-point Likert scale with response options ranging from “strongly disagree” to “strongly agree.” Based on Norman and Skinner [28].

10.2196/89136 Multimedia Appendix 3 eHealth Literacy Scale distribution (N=1525).

10.2196/89136 Multimedia Appendix 4 Odds of low digital health literacy from univariable and multivariable logistic regression models.

10.2196/89136 Multimedia Appendix 5 Odds of low digital health literacy from univariable and multivariable logistic regression models, among participants with a health condition (N=850).

10.2196/89136 Multimedia Appendix 6 Odds of low digital health literacy from univariable and multivariable logistic regression models, using the sample median as an alternative eHealth Literacy Scale cutoff.

10.2196/89136 Multimedia Appendix 7 Odds of low digital health literacy from multivariable logistic regression: results of interaction term testing including age by sex, age by education, and sex by education interaction terms.

10.2196/89136 Multimedia Appendix 8 **.**Odds of low digital health literacy from multivariable logistic regression: complete case analysis versus multiple imputation.

10.2196/89136 Checklist 1 STROBE (Strengthening the Reporting of Observational Studies in Epidemiology)checklist.

## Abbreviations

AIC: Akaike Information Criterion
CCA: complete case analysis
DHL: digital health literacy
eHEALS: eHealth Literacy Scale
MCAR: missing completely at random
MICE: multiple imputation by chained equations
NHS: National Health Service
NRS: National Readership Survey
OR: odds ratio
STROBE: Strengthening the Reporting of Observational Studies in Epidemiology
