# Supplementary material for: Factors Associated With Digital Health Literacy in the United Kingdom: Cross-Sectional Online Survey
Source: J Med Internet Res. 2026 Jul 8;28:e89136. doi: 10.2196/89136 (PMC13345350; doi:10.2196/89136)
Supplement: Multimedia Appendix 1 [file jmir-v28-e89136-s001.doc]

# Multimedia Appendix 1

**Operationaliz**ation of PROGRESS-Plus framework in the survey.

| **PROGRESS-Plus characteristic** | **Survey question** | **Response options** |
| --- | --- | --- |
| **Place of residence** | Which UK region do you live in? | North East  North West  Yorkshire & the Humber  East Midlands  West Midlands  East of England  London  South East  South West  Wales  Scotland  Northern Ireland  Other (please specify) |
|  | Do you live in an urban, suburban or rural area? | Urban  Suburban  Rural |
| **Race/ethnicity/culture/language** | What ethnic group best describes you? | English/Welsh/Scottish/Northern Irish/British  Irish  Gypsy or Irish Traveller  Any other White background  White and Black Caribbean  White and Black African  White and Asian  Any other mixed/multiple ethnic background  Indian  Pakistani  Bangladeshi  Chinese  Any other Asian background  African  Caribbean  Any other Black/African/Caribbean background  Arab  Any other ethnic group  Prefer not to say |
|  | What is the primary language spoken in your household? | English  Polish  Welsh  Portuguese  Arabic  Romanian  Bengali  Spanish  French  Urdu  Gujarati  Chinese  Italian  Panjabi  Other (please specify) |
| **Occupation** | Which of these applies to you? | Working full time (30 or more hours per week)  Working part time (8–29 hours a week)  Working part time (Less than 8 hours a week)  Full time student  Retired  Unemployed  Not working  Other |
| **Gender/sex** | Are you...? | Male  Female |
| **Religion** | Do you regard yourself as belonging to any particular religion, and if so, to which of these do you belong? | No, I do not regard myself as belonging to any particular religion  Yes – Church of England/Anglican/Episcopal  Yes – Roman Catholic  Yes – Presbyterian/Church of Scotland  Yes – Methodist  Yes – Baptist  Yes – Orthodox Christian  Yes – Pentecostal (eg, Assemblies of God, Elim Pentecostal Church, New Testament Church of God, Redeemed Christian Church of God)  Yes – Evangelical – independent/non-denominational (eg, FIEC, Pioneer, Vineyard, Newfrontiers)  Yes – United Reformed Church  Yes – Free Presbyterian  Yes – Brethren  Yes – Judaism  Yes – Hinduism  Yes – Islam  Yes – Sikhism  Yes – Buddhism  Yes – other  Prefer not to say |
| **Education** | What is the highest educational or work-related qualification you have? | No formal qualifications  Youth training certificate/skillseekers  Recognised trade apprenticeship completed  Clerical and commercial  City & Guilds certificate  City & Guilds certificate - advanced  ONC  CSE grades 2–5  CSE grade 1, GCE O level, GCSE, School Certificate  Scottish Ordinary/Lower Certificate  GCE A level or Higher Certificate  Scottish Higher Certificate  Nursing qualification (eg, SEN, SRN, SCM, RGN)  Teaching qualification (not degree)  University diploma  University or CNAA first degree (eg, BA, B.Sc, B.Ed)  University or CNAA higher degree (eg, M.Sc, Ph.D)  Other technical, professional or higher qualification  Don't know  Prefer not to say |
| **Socioeconomic status** | Gross HOUSEHOLD income is the combined income of all those earners in a household from all sources, including wages, salaries, or rents and before tax deductions. What is your gross household income? | Under £5,000 per year  £5,000 to £9,999 per year  £10,000 to £14,999 per year  £15,000 to £19,999 per year  £20,000 to £24,999 per year  £25,000 to £29,999 per year  £30,000 to £34,999 per year  £35,000 to £39,999 per year  £40,000 to £44,999 per year  £45,000 to £49,999 per year  £50,000 to £59,999 per year  £60,000 to £69,999 per year  £70,000 to £99,999 per year  £100,000 to £149,999 per year  £150,000 and over  Don't know  Prefer not to answer |
|  | National Readership Survey social grade [27] | AB  C1  C2  DE |
| **Social capital** | How often, if at all, do you meet with family or friends? | Daily  Weekly  Monthly  Rarely  Never |
| **Age** | What is your age? | 18–34  35–44  45–54  55–64  65+ |
| **Health condition** | Which, if any, of the following chronic conditions have you been diagnosed with by a doctor or healthcare professional (ie, it is expected to last 6 months or more)? Please select all that apply. | Arthritis (osteoarthritis, rheumatoid arthritis, gout, etc.)  Asthma  Cancer  Chronic pain  Chronic obstructive pulmonary disease (COPD), or chronic bronchitis, or emphysema  Type 1 diabetes  Type 2 diabetes  Digestive condition (Irritable Bowel Syndrome (IBS), Gastroesophageal Reflux Disease (GERD/GORD), Crohn's disease, etc.)  Hearing impairment  Heart condition (coronary artery disease, angina, heart failure, etc.)  High blood pressure  High cholesterol  Kidney disease  Mental health condition (anxiety, depression, PTSD, bipolar disorder, eating disorder, etc.)  Migraines  Osteoporosis or osteopenia  Skin condition (acne, eczema, psoriasis, etc.)  Sleep disorder (insomnia, sleep apnea, etc.)  Visual impairment  None of these  Prefer not to say |
| **Disability** | Are your day-to-day activities limited because of a health problem or disability which has lasted, or is expected to last, at least 12 months? | Yes, limited a lot  Yes, limited a little  No |
