# Supplementary material for: Factors Associated With Digital Health Literacy in the United Kingdom: Cross-Sectional Online Survey
Source: J Med Internet Res. 2026 Jul 8;28:e89136. doi: 10.2196/89136 (PMC13345350; doi:10.2196/89136)
Supplement: Multimedia Appendix 2 [file jmir-v28-e89136-s002.doc]

# Multimedia Appendix 2

**eHEALS. Each item is answered on a 5-point Likert scale with response options ranging from “strongly disagree” to “strongly agree”. From Norman and Skinner (2006).**

| **Item** | **Question wording** |
| --- | --- |
| Q1 | I know how to find helpful health resources on the Internet |
| Q2 | I know how to use the Internet to answer my health questions |
| Q3 | I know what health resources are available on the Internet |
| Q4 | I know where to find helpful health resources on the Internet |
| Q5 | I know how to use the health information I find on the Internet to help me |
| Q6 | I have the skills I need to evaluate the health resources I find on the Internet |
| Q7 | I can tell high quality from low quality health resources on the Internet |
| Q8 | I feel confident in using information from the Internet to make health decisions |

Abbreviations: eHEALS, eHealth Literacy Scale
