# Supplementary figures and images for: Factors Associated With Digital Health Literacy in the United Kingdom: Cross-Sectional Online Survey
Source: J Med Internet Res. 2026 Jul 8;28:e89136. doi: 10.2196/89136 (PMC13345350; doi:10.2196/89136)

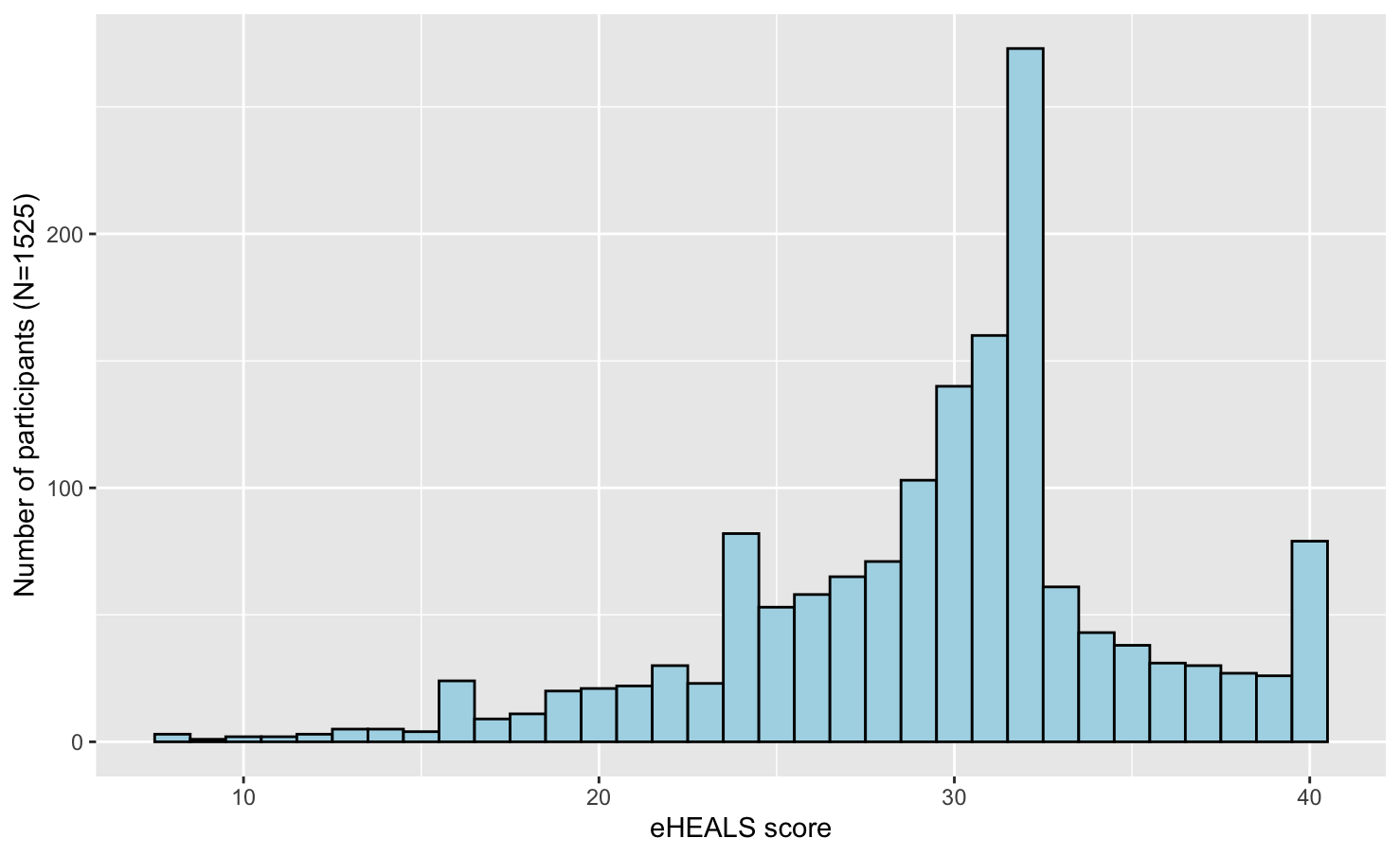

Supplement: Multimedia Appendix 3 [file jmir-v28-e89136-s003.png]
