# Supplementary material for: Factors Associated With Digital Health Literacy in the United Kingdom: Cross-Sectional Online Survey
Source: J Med Internet Res. 2026 Jul 8;28:e89136. doi: 10.2196/89136 (PMC13345350; doi:10.2196/89136)
Supplement: Multimedia Appendix 4 [file jmir-v28-e89136-s004.docx]

# Multimedia Appendix 4

**Odds of low DHL from univariable and multivariable logistic regression models.**

| Variable | Univariable models: Unadjusted OR (95% CI) | Multivariable model:  Adjusted OR (95% CI) ^a^ |
| --- | --- | --- |
| UK region  England  Wales  Scotland  Northern Ireland | [Reference]  1.04 (0.60-1.80)  1.32 (0.87-1.99)  1.56 (0.68-3.59) | N/A |
| Urbanicity ^b^  Nonurban  Urban | [Reference]  0.90 (0.67-1.23) | [Reference]  0.93 (0.68-1.28) |
| Ethnicity  White  Other | [Reference]  0.85 (0.57-1.27) | [Reference]  1.00 (0.64-1.56) |
| Primary language  English  Other | [Reference]  1.02 (0.55-1.91) | N/A |
| Employment status  Working  Student  Retired  Unemployed/not working  Other | [Reference]  1.21 (0.59-2.51)  1.23 (0.92-1.65)  0.89 (0.57-1.38)  1.31 (0.76-2.26) | N/A |
| Sex  Male  Female | [Reference]  0.59 (0.46-0.76) *** | [Reference]  0.60 (0.46-0.77) *** |
| Religion  No  Yes | [Reference]  0.86 (0.67-1.11) | [Reference]  0.81 (0.62-1.06) |
| Educational attainment  Below degree-level  Undergraduate degree  Postgraduate degree or higher | [Reference]  0.46 (0.33-0.64) ***  0.54 (0.38-0.75) *** | [Reference]  0.52 (0.37-0.74) ***  0.58 (0.40-0.82) ** |
| Social grade  ABC1  C2DE | [Reference]  1.66 (1.29-2.13) *** | [Reference]  1.37 (1.05-1.80) * |
| Annual household income  Less than £20,000  £20,000-£39,999  £40,000-£59,999  £60,000 or greater | [Reference]  0.81 (0.58-1.12)  0.69 (0.45-1.03)  0.64 (0.42-0.97) * | N/A |
| Frequency of meeting with family or friends  Never or rarely  Weekly or monthly  Daily | [Reference]  0.67 (0.47-0.97) *  0.55 (0.36-0.83) ** | [Reference]  0.77 (0.53-1.12)  0.64 (0.42-1.00) * |
| Age group  18-44 years  45-64 years  65 years and older | [Reference]  1.29 (0.96-1.72)  1.56 (1.15-2.13) ** | [Reference]  1.21 (0.89-1.65)  1.43 (1.02-2.01) * |
| Health condition  No  Yes | [Reference]  0.92 (0.71-1.19) | N/A |
| Limited activity  No  Yes | [Reference]  1.01 (0.77-1.33) | [Reference]  0.84 (0.63-1.13) |

Abbreviations: DHL, digital health literacy; OR, odds ratio; CI, confidence interval; UK, United Kingdom

^a^ Built using enter method, predictor variables omitted after investigation of associations; ^b^ Participants were asked “Do you live in an urban, suburban or rural area?” with answer options urban, suburban, rural; this variable was dichotomized to give urban and nonurban as groups.

* Significant at p < 0.05, ** Significant at p < 0.01, *** Significant at p < 0.001
