# Supplementary material for: Factors Associated With Digital Health Literacy in the United Kingdom: Cross-Sectional Online Survey
Source: J Med Internet Res. 2026 Jul 8;28:e89136. doi: 10.2196/89136 (PMC13345350; doi:10.2196/89136)
Supplement: Multimedia Appendix 5 [file jmir-v28-e89136-s005.docx]

# Multimedia Appendix 5

**Odds of low DHL from univariable and multivariable logistic regression models, among participants with a health condition (N=850).**

| **Variable** | **Univariable models: Unadjusted OR (95% CI)** | **Multivariable model:**  **Adjusted OR (95% CI) ^a^** |
| --- | --- | --- |
| **UK region**  England  Wales  Scotland  Northern Ireland | [Reference]  1.11 (0.56-2.20)  1.13 (0.66-1.95)  0.81 (0.24-2.72) | N/A |
| **Urbanicity** ^b^  Nonurban  Urban | [Reference]  1.06 (0.71-1.59) | [Reference]  1.04 (0.69-1.60) |
| **Ethnicity**  White  Other | [Reference]  1.25 (0.69-2.27) | [Reference]  1.55 (0.80-3.05) |
| **Primary language**  English  Other | [Reference]  1.55 (0.69-3.48) | N/A |
| **Employment status**  Working  Student  Retired  Unemployed/not working  Other | [Reference]  0.57 (0.08-4.10)  1.39 (0.97-2.01)  1.11 (0.63-1.97)  1.47 (0.72-3.02) | N/A |
| **Sex**  Male  Female | [Reference]  0.55 (0.39-0.77) *** | [Reference]  0.58 (0.41-0.83) ** |
| **Religion**  No  Yes | [Reference]  0.99 (0.71-1.39) | [Reference]  0.88 (0.61-1.26) |
| **Educational attainment**  Below degree-level  Undergraduate degree  Postgraduate degree or higher | [Reference]  0.27 (0.16-0.48) ***  0.48 (0.30-0.75) ** | [Reference]  0.30 (0.16-0.54) ***  0.52 (0.32-0.83) ** |
| **Social grade**  ABC1  C2DE | [Reference]  1.84 (1.31-2.58) *** | [Reference]  1.41 (0.97-2.04) |
| **Annual household income**  Less than £20,000  £20,000-£39,999  £40,000-£59,999  £60,000 or greater | [Reference]  0.75 (0.49-1.13)  0.61 (0.36-1.04)  0.50 (0.27-0.93) * | N/A |
| **Frequency of meeting with family or friends**  Never or rarely  Weekly or monthly  Daily | [Reference]  0.60 (0.37-0.97) *  0.45 (0.25-0.80) ** | [Reference]  0.68 (0.41-1.13)  0.53 (0.29-0.96) * |
| **Age group**  18-44 years  45-64 years  65 years and older | [Reference]  1.07 (0.70-1.66)  1.40 (0.92-2.14) | [Reference]  0.92 (0.58-1.46)  1.18 (0.74-1.88) |
| **Health condition**  No  Yes | N/A | N/A |
| **Limited activity**  No  Yes | [Reference]  1.08 (0.78-1.50) | [Reference]  0.93 (0.66-1.33) |

Abbreviations: DHL, digital health literacy; OR, odds ratio; CI, confidence interval; UK, United Kingdom; N/A, not applicable

^a^ Built using enter method, predictor variables omitted after investigation of associations; ^b^ Participants were asked “Do you live in an urban, suburban or rural area?” with answer options urban, suburban, rural; this variable was dichotomized to give urban and nonurban as groups.

* Significant at *P*<.05, ** Significant at *P*<.01, *** Significant at *P*<.001
