# Supplementary material for: Factors Associated With Digital Health Literacy in the United Kingdom: Cross-Sectional Online Survey
Source: J Med Internet Res. 2026 Jul 8;28:e89136. doi: 10.2196/89136 (PMC13345350; doi:10.2196/89136)
Supplement: Multimedia Appendix 6 [file jmir-v28-e89136-s006.doc]

# Multimedia Appendix 6

**Odds of low DHL from univariable and multivariable logistic regression models, using the sample median as an alternative eHEALS cutoff.**

| **Variable** | **Univariable models: Unadjusted OR (95% CI)** | **Multivariable model:**  **Adjusted OR (95% CI) ^a^** |
| --- | --- | --- |
| **UK region**  England  Wales  Scotland  Northern Ireland | [Reference]  1.68 (1.06-2.66)  0.97 (0.68-1.38)  1.04 (0.49-2.20) | N/A |
| **Urbanicity** ^b^  Nonurban  Urban | [Reference]  0.96 (0.75-1.24) | [Reference]  1.02 (0.79-1.32) |
| **Ethnicity**  White  Other | [Reference]  0.89 (0.65-1.22) | [Reference]  1.08 (0.76-1.54) |
| **Primary language**  English  Other | [Reference]  1.84 (1.08-3.14) | N/A |
| **Employment status**  Working  Student  Retired  Unemployed/not working  Other | [Reference]  1.43 (0.77-2.65)  1.41 (1.10-1.79) **  1.05 (0.74-1.47)  1.29 (0.81-2.06) | N/A |
| **Sex**  Male  Female | [Reference]  0.70 (0.57-0.86) *** | [Reference]  0.70 (0.57-0.86) *** |
| **Religion**  No  Yes | [Reference]  0.88 (0.72-1.10) | [Reference]  0.81 (0.65-1.01) |
| **Educational attainment**  Below degree-level  Undergraduate degree  Postgraduate degree or higher | [Reference]  0.53 (0.41-0.68) ***  0.46 (0.35-0.60) *** | [Reference]  0.60 (0.46-0.78) ***  0.49 (0.37-0.65) *** |
| **Social grade**  ABC1  C2DE | [Reference]  1.60 (1.31-1.96) *** | [Reference]  1.32 (1.05-1.64) * |
| **Annual household income**  Less than £20,000  £20,000-£39,999  £40,000-£59,999  £60,000 or greater | [Reference]  0.90 (0.67-1.21)  0.79 (0.57-1.12)  0.58 (0.41-0.81) ** | N/A |
| **Frequency of meeting with family or friends**  Never or rarely  Weekly or monthly  Daily | [Reference]  0.73 (0.53-1.01)  0.61 (0.42-0.87) ** | [Reference]  0.80 (0.57-1.12)  0.68 (0.47-1.00) * |
| **Age group**  18-44 years  45-64 years  65 years and older | [Reference]  1.41 (1.12-1.78) **  1.66 (1.28-2.15) *** | [Reference]  1.38 (1.07-1.76) *  1.59 (1.20-2.11) ** |
| **Health condition**  No  Yes | [Reference]  1.00 (0.82-1.24) | N/A |
| **Limited activity**  No  Yes | [Reference]  1.01 (0.81-1.26) | [Reference]  0.83 (0.65-1.05) |

Abbreviations: DHL, digital health literacy; OR, odds ratio; CI, confidence interval; UK, United Kingdom; N/A, not applicable

^a^ Built using enter method, predictor variables omitted after investigation of associations; ^b^ Participants were asked “Do you live in an urban, suburban or rural area?” with answer options urban, suburban, rural; this variable was dichotomized to give urban and nonurban as groups.

* Significant at *P*<.05, ** Significant at *P*<.01, *** Significant at *P*<.001
