# Supplementary material for: Factors Associated With Digital Health Literacy in the United Kingdom: Cross-Sectional Online Survey
Source: J Med Internet Res. 2026 Jul 8;28:e89136. doi: 10.2196/89136 (PMC13345350; doi:10.2196/89136)
Supplement: Multimedia Appendix 7 [file jmir-v28-e89136-s007.docx]

# Multimedia Appendix 7

**Odds of low DHL from multivariable logistic regression: results of interaction term testing including age by sex, age by education, and sex by education interaction terms.**

| Interaction term | Adjusted OR (95% CI) | P-value |
| --- | --- | --- |
| Age by sex  Female x 45-64 years  Female x 65 years and older | 0.44 (0.24-0.81)  0.80 (0.42-1.50) | .009  .48 |
| Age by education  45-64 years x Undergraduate degree  45-64 years x Postgraduate degree or higher  65 years and older x Undergraduate degree  65 years and older x Postgraduate degree or higher | 1.01 (0.47-2.17)  0.72 (0.30-1.73)  1.04 (0.42-2.58)  1.73 (0.75-4.01) | .97  .46  .94  .20 |
| Sex by education  Female x Undergraduate degree  Female x Postgraduate degree or higher | 1.42 (0.73-2.77)  0.77 (0.37-1.60) | .31  .48 |

Abbreviations: DHL, digital health literacy; OR, odds ratio; CI, confidence interval

ORs and 95% CIs are presented for interaction terms only. All models additionally include the main effects of urbanicity, ethnicity, sex, religion, education, social grade, frequency of socialising, age group, and limited activity.

Multiple imputation was based on the full sample with 20 imputed datasets pooled using Rubin's rules.

Akaike Information Criterion (AIC) values for the complete case interaction models were: primary model = 1258.8; age x sex = 1256.8; age x education = 1261.6; sex x education = 1260.1.
