# Supplementary material for: Factors Associated With Digital Health Literacy in the United Kingdom: Cross-Sectional Online Survey
Source: J Med Internet Res. 2026 Jul 8;28:e89136. doi: 10.2196/89136 (PMC13345350; doi:10.2196/89136)
Supplement: Multimedia Appendix 8 [file jmir-v28-e89136-s008.docx]

# Multimedia Appendix 8

**Odds of low DHL from multivariable logistic regression: complete case analysis versus multiple imputation.**

| Variable | Complete case analysis multivariable model ^a^ | | Multiple imputation multivariable model ^a^ | |
| --- | --- | --- | --- | --- |
|  | **Adjusted OR (95% CI)** | **Standard errors** | **Adjusted OR (95% CI)** | **Standard errors** |
| UK region  England  Wales  Scotland  Northern Ireland | N/A | N/A | N/A | N/A |
| Urbanicity ^b^  Nonurban  Urban | [Reference]  0.94 (0.68-1.33) | [Reference]  0.17 | [Reference]  0.93 (0.68-1.28) | [Reference]  0.16 |
| Ethnicity  White  Other | [Reference]  1.10 (0.63-1.83) | [Reference]  0.27 | [Reference]  1.00 (0.64-1.56) | [Reference]  0.23 |
| Primary language  English  Other | N/A | N/A | N/A | N/A |
| Employment status  Working  Student  Retired  Unemployed/not working  Other | N/A | N/A | N/A | N/A |
| Sex  Male  Female | [Reference]  0.55 (0.42-0.74) *** | [Reference]  0.15 | [Reference]  0.60 (0.46-0.77) *** | [Reference]  0.13 |
| Religion  No  Yes | [Reference]  0.72 (0.53-0.97) * | [Reference]  0.15 | [Reference]  0.81 (0.62-1.06) | [Reference]  0.14 |
| Educational attainment  Below degree-level  Undergraduate degree  Postgraduate degree or higher | [Reference]  0.49 (0.33-0.71) ***  0.48 (0.32-0.71) *** | [Reference]  0.19  0.20 | [Reference]  0.52 (0.37-0.74) ***  0.58 (0.40-0.82) ** | [Reference]  0.18  0.18 |
| Social grade  ABC1  C2DE | [Reference]  1.31 (0.97-1.77) | [Reference]  0.15 | [Reference]  1.37 (1.05-1.80) * | [Reference]  0.14 |
| Annual household income  Less than £20,000  £20,000-£39,999  £40,000-£59,999  £60,000 or greater | N/A | N/A | N/A | N/A |
| Frequency of meeting with family or friends  Never or rarely  Weekly or monthly  Daily | [Reference]  0.82 (0.54-1.27)  0.65 (0.40-1.07) | [Reference]  0.22  0.25 | [Reference]  0.77 (0.53-1.12)  0.64 (0.42-1.00) * | [Reference]  0.19  0.22 |
| Age group  18-44 years  45-64 years  65 years and older | [Reference]  1.61 (1.13-2.31) **  1.98 (1.36-2.91) *** | [Reference]  0.18  0.19 | [Reference]  1.21 (0.89-1.65)  1.43 (1.02-2.01) * | [Reference]  0.16  0.17 |
| Health condition  No  Yes | N/A | N/A | N/A | N/A |
| Limited activity  No  Yes | [Reference]  0.87 (0.63-1.18) | [Reference]  0.16 | [Reference]  0.84 (0.63-1.13) | [Reference]  0.15 |

Abbreviations: DHL, digital health literacy; OR, odds ratio; CI, confidence interval; UK, United Kingdom, N/A, not applicable

Complete case analysis was based on n=1,327; multiple imputation was based on the full sample with 20 imputed datasets pooled using Rubin's rules.

^a^ Built using enter method, predictor variables omitted after investigation of associations; ^b^ Participants were asked “Do you live in an urban, suburban or rural area?” with answer options urban, suburban, rural; this variable was dichotomized to give urban and nonurban as groups.

* Significant at p < 0.05, ** Significant at p < 0.01, *** Significant at p < 0.001
